# Jujube (*Ziziphus jujuba* Mill.) Protects Hepatocytes against Alcohol-Induced Damage through Nrf2 Activation

**DOI:** 10.1155/2020/6684331

**Published:** 2020-12-24

**Authors:** Sungwha Hong, Younghwa Kim, Jeehye Sung, Hana Lee, Huijin Heo, Heon Sang Jeong, Junsoo Lee

**Affiliations:** ^1^Department of Food Science and Biotechnology, Chungbuk National University, Cheongju, Chungbuk 28644, Republic of Korea; ^2^School of Food Biotechnology and Nutrition, Kyungsung University, Busan 48434, Republic of Korea; ^3^Food Science and Biotechnology, Andong National University, Andong, Gyeongbuk 36729, Republic of Korea

## Abstract

This study aimed at evaluating the cytoprotective activity of jujube water extract (JWE) against alcohol-induced oxidative stress via the activation of the Nrf2 pathway in HepG2 cells. JWE had various phenolic compounds, and the vanillic acid content was the highest in the extract. To determine the cytoprotective effect of JWE against alcohol-induced damage, hepatocytes were treated with JWE and 3% ethanol. JWE (100 *μ*g/mL) markedly increased cell viability by approximately 100% in a dose-dependent manner. Moreover, JWE attenuated the production of malondialdehyde, reactive oxygen species, aspartate, and alanine aminotransferase and the depletion of glutathione. Moreover, JWE enhanced the expression of antioxidant defense enzymes including heme oxygenase-1, NADPH quinone oxidoreductase 1, and *γ*-glutamate-cysteine ligase catalytic against alcohol-induced oxidative damage in hepatocytes via the activation of Nrf2. Taken together, JWE possesses the protective effect against alcohol-induced oxidative injury in hepatocytes through the upregulation of the Nrf2 signaling pathway. Therefore, jujube fruit might have the potential to improve alcohol-related liver problems.

## 1. Introduction

Overconsumption of alcohol leads to alcohol-related liver disease (ALD). Besides ALD, alcohol is also a major cause of mortality and morbidity [1]. ALD becomes a major global health problem due to the increasing alcohol consumption [2]. Ingested alcohol is detoxified principally in the liver by converting the alcohol into acetaldehyde using alcohol dehydrogenase [3]. During chronic alcohol consumption, cytochrome p450 2E1 (CYP2E1) could be induced and might play a pivotal role in alcohol metabolism [4]. CYP2E1 is well known as a potential source of reactive oxygen species (ROS) during alcohol oxidation and depletes cellular glutathione (GSH) [5, 6]. In addition, it was reported that excessive ROS generation causes liver damage by inducing proinflammatory cytokine production [7, 8]. Therefore, attempting to lower ROS levels might be an effective strategy in ALD treatment and prevention.

Nuclear factor erythroid-2-related factor 2 (Nrf2) is a key protein in antioxidant signaling against oxidative damage via inducing the expression of antioxidant genes. Nrf2 in the cytoplasm translocates into the nucleus and binds to the antioxidant response element (ARE) in the upstream promoter region of various antioxidant genes including NAD(P)H quinone oxidoreductase 1 (NQO1), heme oxygenase-1 (HO-1), and gamma glutamate-cysteine ligase (*γ*-GCLC) [9]. In a previous report, the activated Nrf2/ARE pathway enhanced cytoprotection against alcohol stress and ameliorated ALD [10]. In contrast, Nrf2 knockdown caused hepatocyte necroptosis against alcohol stress [11]. Based on the various pieces of evidence, Nrf2 activation is generally considered as a protective response against alcohol-induced damage.

Jujube (*Ziziphus jujuba* Mill.) is broadly distributed in Asia and Europe [12]. Jujube fruit is especially rich in minerals, sugars, organic acids, volatile compounds, and fibers [13]. Jujube also contains various phenolic acids [14] and flavonoids [15], responsible for its health benefits. In previous studies, jujube fruit showed various biological activities including anticancer [16], antioxidant [17], and anti-inflammatory [18] activities. Despite previous results concerning the hepatoprotective effects of jujube [19], a more detailed description is still needed to clarify the effector mechanism behind the protective activity of jujube in hepatocytes against alcohol-induced oxidative damage. This study aimed at investigating this subject. Moreover, the protective mechanism through the activation of the Nrf2 signaling pathway was studied in human hepatocytes.

## 2. Materials and Methods

### 2.1. Jujube Water Extract

Jujube flesh was washed, lyophilized, and ground into fine powder. For jujube water extract (JWE), the powder (20 g) was added to 400 mL of water, and then this mixture was shaken mechanically for 24 h and filtered (Advantec, No. 2, Toyo Roshi, Tokyo, Japan). The JWE was concentrated by freeze-drying and redissolved in distilled water.

### 2.2. Standard Solution and Sample Preparation

All flavonoid standard solutions were prepared in methanol. The JWE (0.05 g) was mixed with methanol (500 *μ*L) containing internal standards (salicylic acid-d_6_ and apigenin-d_5_). The suspensions were vortexed for 20 min at room temperature and extracted by sonication (40 kHz) for 20 min. The methanol extract of the stalk end of persimmon was diluted with methanol containing internal standards. After centrifugation at 3,000 ×g for 10 min at 4°C, it was filtered through syringe filters (0.22 *μ*m nylon filter, Bonna-Agela Technologies Inc., Wilmington, NC).

### 2.3. LC-MS/MS Analysis

The LC-MS/MS analysis was performed as described previously [20]. In brief, analytes were chromatographed on the column (Acclaim C30, 150 mm × 2.1 mm, 3.0 *μ*m particle size, Thermo Fisher Scientific, San Jose, CA, USA) at 25°C. An external standard method for phenolic acids and flavonoids was used for the quantitation of the analytes of interest. Data analysis was performed using the Xcalibur v3.0 (Thermo Fisher Scientific).

### 2.4. Cell Culture and Cytotoxicity

HepG2 cells were purchased from the Korean Collection for Type Cultures (Daejeon, Korea). The cells were maintained in Dulbecco's Modified Eagle Medium (Gibco BRL, Gaithersburg, MD, USA) containing 10% heat-inactivated FBS (Gibco BRL), 100 U/mL penicillin, and 100 *μ*g/mL streptomycin in a humidified 5% CO_2_ incubator at 37°C.

Cell viability was measured by MTT assay. To measure the cytotoxicity of ethanol and/or JWE, the cells were exposed to ethanol (from 1% to 4%) and/or JWE (25, 50, and 100 *μ*g/mL) for 24 h. Moreover, to evaluate the cytoprotection of JWE against ethanol treatment, HepG2 cells were treated with an FBS-free medium containing ethanol (3%) and different concentrations of JWE for 24 h. The upcoming day, MTT reagent (0.5 mg/mL) was treated for 2 h. The color intensity of the formazan was measured spectrophotometrically at 550 nm.

### 2.5. Quantification of ROS Generation

2′,7′-Dichlorofluorescin diacetate (DCF-DA) fluorescent probe was used for the quantification of intracellular ROS as described previously [21]. The fluorescence intensity was evaluated at 485 nm (an excitation wavelength) and 530 nm (an emission wavelength) using a fluorescence spectrophotometer.

### 2.6. Determination of Glutathione and Lipid Peroxidation

HepG2 cells were exposed to JWE and 3% ethanol for 24 h, and then cells were harvested to measure the GSH and malondialdehyde (MDA) levels. Intracellular GSH levels were determined as described previously with slight modification [22]. To determine the cellular lipid peroxidation, the thiobarbituric acid reactive substances assay was conducted [23].

### 2.7. Levels of ALT and AST

The levels of alanine aminotransferase (ALT) and aspartate aminotransferase (AST) in HepG2 cells were evaluated using assay kit (Biovision, CA, USA).

### 2.8. Western Blotting

Western blotting analysis was examined to evaluate the expression levels of antioxidant-related proteins as described previously [9]. In brief, HepG2 cells were exposed to different concentrations of JWE (25, 50, and 100 *μ*g/mL) and 3% ethanol for 3 h, rinsed with PBS, and collected by centrifugation. The equal quantities of proteins (50 *μ*g for lane) were loaded on 10% sodium dodecyl sulfate-polyacrylamide gel and transferred onto nitrocellulose membranes (Hybond-Nb, Amersham Pharmacia, GE Healthcare, Bukinghamshire, UK). The membranes were blocked in 5% skim milk for 1 h and then incubated in blocking solution with *γ*-GCLC, NQO-1, HO-1, *β*-actin, PCNA, and Nrf2 for 1 h at room temperature. After washing in Tris-buffered saline/Tween 20 (TBST), the membranes were exposed to horseradish-conjugated secondary anti-rabbit or anti-mouse antibodies for 1 h at room temperature. ECL™ reagent (GE Healthcare, Buckinghamshire, UK) was used to detect the protein signal intensity. The density of the protein band was quantified using image software (LabImage 1D program, Kapelan Bio-Imaging, Halle/Saale, Germany).

## 3. Results and Discussion

### 3.1. The Composition of Phytochemicals in Jujube Water Extract

To examine the biological activity of jujube, lyophilized jujube fruit except for its seed was extracted with water (44.61% extraction yield, w/w). Phytochemicals including flavonoids and phenolic acids are responsible for various biological activities and health benefits. It was reported that the antioxidant capacity of jujube correlated well with the content of its bioactive compounds such as polyphenols and flavonoids [24]. The composition of TWE phenolic acids and flavonoids were evaluated using LC-MS/MS (Table 1). Sixteen different phenolic compounds were screened for identification and quantification. In the results, six phenolic acids (caffeic acid, coumaric acid, sinapic acid, vanillic acid, ferulic acid, and benzoic acid) and three flavonoids (taxifolin, phloridzin, and rutin) were successfully identified and measured in JWE. In particular, the content of vanillic acid (800.71 *μ*g/g JWE) was the highest of phenolic compounds in JWE. Benzoic acid and rutin also represented markedly high phenolic contents (456.25 *μ*g/g JWE and 226.06 *μ*g/g JWE, respectively). It has been previously reported that 5 phenolic components (catechol, p-coumaric acid, p-hydroxybenzoic acid, protocatechuic acid, and vanilla acid) were identified in the extract [19]. Another study reported the identification of quercetin and kaempferol derivatives from ethanolic jujube extracts [25]. In this study, we found and measured 9 phenolics in jujube, including caffeic acid, coumaric acid, sinapic acid, vanillic acid, ferulic acid, benzoic acid, taxifolin, phloridzin, and rutin. Therefore, JWE could possess several beneficial effects on human health due to its various phenolic compounds.

### 3.2. Cytoprotective Effects of Jujube Water Extract against Alcohol Damage in HepG2 Cells

**Figure 1:**
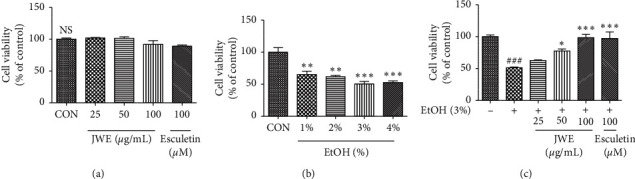
Cytotoxic effect of jujube water extract and ethanol in HepG2 cells. Cells were treated with indicated concentrations of jujube water extract (a) and ethanol (b) for 24 h. The protective effect of jujube water extract against alcohol stress was also measured (c). ^###^*p* < 0.001, significant difference compared with the control cells, ^*∗∗∗*^*p* < 0.001 and ^*∗∗*^*p* < 0.01, significant difference compared with the control cells. NS, not significant; JWE, jujube water extract; EtOH, ethanol.

**Figure 2:**
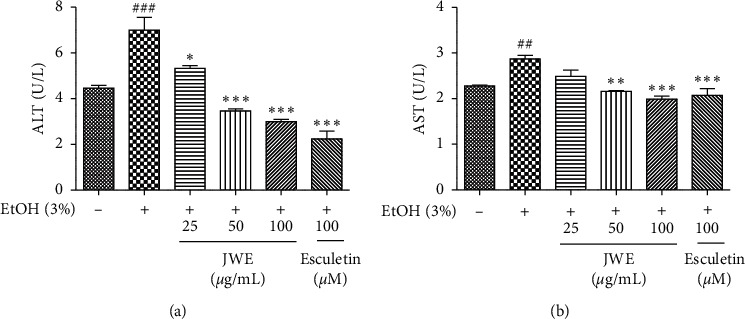
Effects of jujube water extract on cellular ALT (a) and AST (b) levels against alcohol stress in HepG2 cells. ^###^*p* < 0.001 and ^##^*p* < 0.01, significant difference compared with the control cells, ^*∗∗∗*^*p* < 0.001, ^*∗∗*^*p* < 0.01, and ^*∗*^*p* < 0.05, significant difference compared with the ethanol-treated group. JWE, jujube water extract; EtOH, ethanol; AST, aspartate aminotransferase; ALT, alanine aminotransferase.

As shown in Figure 1(a), JWE (25, 50, and 100 *μ*g/mL) and esculetin (100 *μ*M) did not show cytotoxic effects in HepG2 cells. The treatment of ethanol (1, 2, 3, and 4%, w/w) decreased cell viability, and 3% ethanol treatment induced a 40% cytotoxic effect in HepG2 cells (Figure 1(b)). To investigate the cytoprotective effects of JWE against alcohol damage, the cells were treated with JWE and 3% ethanol for 24 h. The results showed that JWE dose-dependently reduced cell death against ethanol-induced oxidative damage in hepatocytes (Figure 1(c)). Alanine aminotransferase (ALT) and aspartate aminotransferase (AST) have become the most important markers of liver injury [26]. In the present study, the levels of ALT and AST in HepG2 cells were determined to assess the ethanol-induced liver damage (Figures 2(a) and 2(b)). We observed that the treatment with 3% ethanol significantly increased ALT and AST levels compared with the control untreated cells. However, the treatment with JWE decreased the ALT and AST levels in a concentration-dependent manner. Therefore, these results suggest that JWE ameliorated the alcoholic liver injury. Previously, it was reported that vanillic acid, benzoic acid, and rutin had protective effects in the case of liver injury [27, 28]. In particular, a previous study showed that several flavonoids in jujube played a hepatoprotective role against acetaminophen-induced liver injury [25]. Therefore, these results indicated that the cytoprotective effects of JWE might be associated with its phenolic acids and flavonoids, and jujube fruit could exhibit therapeutic potential in liver damage treatment.

### 3.3. Antioxidative Effects of Jujube Water Extract against Alcohol-Induced Oxidative Stress

**Figure 3:**
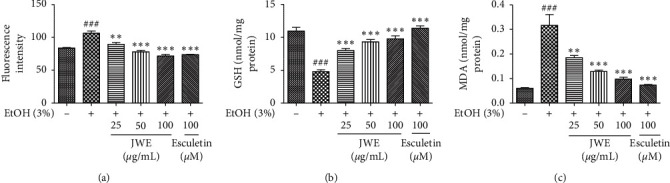
Effects of jujube water extract on the generation of reactive oxygen spices (a), glutathione (b), and malondialdehyde (c) against alcohol stress in HepG2 cell. ^###^*p* < 0.001, significant difference compared with the control cells, ^*∗∗∗*^*p* < 0.001 and ^*∗∗*^*p* < 0.01, significant difference compared with the ethanol-treated group. JWE, jujube water extract; EtOH, ethanol; GSH, glutathione; MDA, malondialdehyde.

Alcohol-induced oxidative stress might contribute to the exhaustion of antioxidant capabilities in the liver [29]. Moreover, intracellular ROS overproduction was associated with certain pathological conditions, including liver diseases [30]. To determine the antioxidative effects of JWE against alcohol-induced oxidative stress, it was conducted using DCF-DA fluorescence assay, GSH assay, and MDA assay in HepG2 cells. The exposure to ethanol (3%) significantly increased ROS generation compared with the control (Figure 3(a)), while the JWE treatment significantly inhibited it. In addition, alcohol metabolism via CYP2E1 leads to ROS overproduction and the depletion of reduced GSH in the liver, which might promote lipid peroxidation [31]. GSH plays an important role in the cellular antioxidative defense system in mammalian cells [32]. Therefore, we examined the effect of JWE on the GSH depletion against alcohol stress.  Figure 3(b) shows that exposure to ethanol decreased the intracellular GSH level compared with the control, whereas treatment with JWE significantly increased the GSH level against ethanol-induced oxidative damage in hepatocytes. Next, we also measured the MDA levels in HepG2 cells (Figure 3(c)). A 24-hour exposure to ethanol significantly increased the intracellular MDA level compared to the control. However, the JWE treatment (100 *μ*g/mL) decreased the MDA levels by approximately 70%. In a previous study, liver damage by acetaminophen at a toxic dose induced the increase of GSH exhaustion, lipid peroxidation, and inflammation [33]. Taken together, the JWE treatment dramatically suppressed ROS generation, lipid peroxidation, and GSH depletion against ethanol-induced oxidative damage stress in HepG2 cells. These results clearly showed that JWE could allow to restore a normal redox status of cells and to ameliorate the cytotoxicity against alcohol-induced oxidative stress in hepatocytes.

### 3.4. Effects of Jujube Water Extract on the Expression of Phase II Enzymes and the Activation of Nrf2

**Figure 4:**
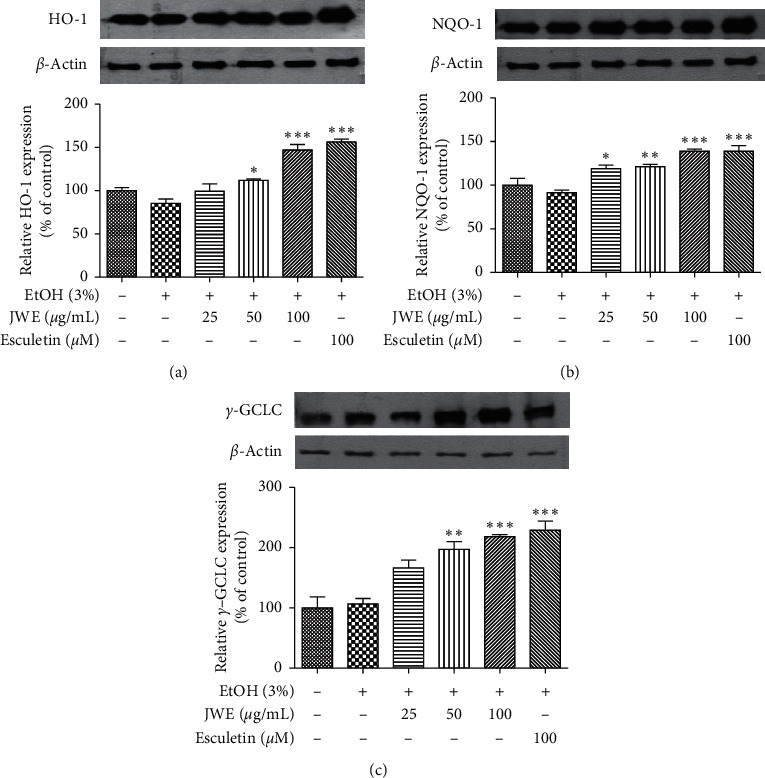
Effects of jujube water extract on HO-1 (a), NQO-1 (b), and GCLC (c) protein expressions against alcohol stress in HepG2 cells. ^*∗∗∗*^*p* < 0.001, ^*∗∗*^*p* < 0.01, and ^*∗*^*p* < 0.05, significant difference compared with the ethanol-treated group. JWE, jujube water extract; EtOH, ethanol.

To elucidate the cytoprotective mechanism of JWE against alcohol-induced oxidative damage, HepG2 cells were exposed to JWE (25, 50, and 100 *μ*g/mL) and ethanol (3%) for 24 h. The results indicated that the JWE treatment fully protected the HepG2 cells against ethanol-induced oxidative damage. It has been already well known that antioxidant enzymes play a critical role in ethanol-induced oxidative stress in hepatocytes [34]. HO-1, NQO1, and GCLC are good indicators for the induction of an adaptive response to several stimulations, including oxidative stress [35, 36]. To elucidate the protective mechanism of JWE in HepG2 cells, we thus examined the protein expressions of antioxidant enzymes including NQO1, GCLC, and HO-1. As shown in Figure 4, the JWE treatment induced and increased the protein expressions of NQO1, HO-1, and GCLC in a dose-dependent manner. Previously, it was reported that naturally occurring antioxidants increased the expression of antioxidant enzymes such as NQO1, HO-1, and GCLC [37]. In addition, several flavonoids increase the level of glutathione by the transactivation of the catalytic subunit promoter of GCLC [38].

**Figure 5:**
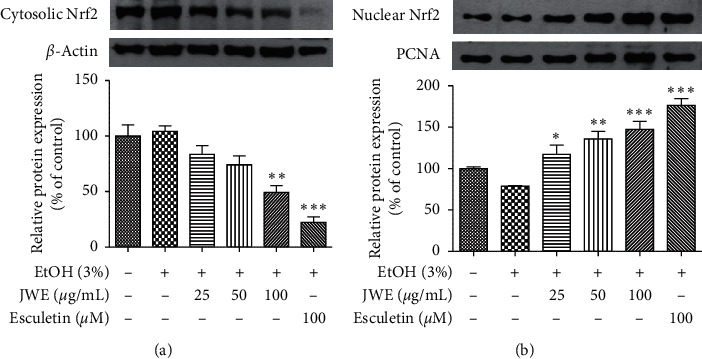
Effects of jujube water extract on the Nrf2 protein expressions in the cytosol (a) and nucleus (b) against alcohol stress in HepG2-cells. ^*∗∗∗*^*p* < 0.001, ^*∗∗*^*p* < 0.01, and ^*∗*^*p* < 0.05, significant difference compared with the ethanol-treated group. JWE, jujube water extract; EtOH, ethanol.

Most antioxidant and detoxifying enzymes have an ARE sequence in their promoter region [39]. Nrf2 is a critical transcription factor regulating ARE-driven NQO1, HO-1, and GCLC gene expression [40]. In this study, we also evaluated the JWE-induced activation of Nrf2 in HepG2 cells. The JWE treatment significantly decreased the cytosolic Nrf2 protein expression in a dose-dependent manner (Figure 5(a)). Moreover, the JWE treatment significantly (*p* < 0.05) increased the nuclear Nrf2 protein expression, when compared with the control group (Figure 5(b)). It is well known that Nrf2 dissociates from Keap1 during stimulation, and then it translocates from the cytosol into the nucleus [41]. The Nrf2/ARE pathway plays a critical role in the protective effect against various stresses including chemicals, inflammatory agents, metals, and alcohol [42]. Especially, quercetin, a plant flavonoid, showed ethanol toxicity and HO-1 induction via Nrf2 activation in hepatocytes [43]. Phenolic acid also decreased hepatocytes damage against oxidative stress in HepG2 cells [44]. These results indicate that JWE induced the Nrf2 activation and upregulation of phase II enzyme against alcohol stress. Moreover, these results suggest that the JWE-mediated cytoprotection against alcohol stress could be associated with the antioxidative activities of its phenolic compounds in the extract.

## 4. Conclusions

In conclusion, JWE showed cytoprotective effects against alcohol-induced oxidative stress in hepatocytes. JWE contained various phenolics, and vanillic acid was the most abundant component. The JWE treatment increased cell viability against alcohol treatment and reduced the AST and ALT levels. JWE also effectively inhibited GSH depletion, ROS production, and lipid peroxidation against ethanol-induced oxidative injury in hepatocytes. Furthermore, the treatment of JWE activated the Nrf2 pathway and induced the protein expression of antioxidant defense enzymes against alcohol stress. Taken together, these results suggest that JWE might have potential as a functional ingredient for attenuating ethanol-induced liver damage.

## Figures and Tables

**Table 1:** Contents of phenolic acids and flavonoids in jujube water extract.

Phenolic acids and flavonoids	Concentration (*μ*g/g jujube water extract)
Caffeic acid	52.72 ± 1.90
Coumaric acid	153.35 ± 69.91
Sinapic acid	26.57 ± 0.60
Gallic acid	ND^1^
Vanillic acid	800.71 ± 195.15
Ferulic acid	77.02 ± 10.48
Benzoic acid	456.25 ± 27.33
Taxifolin	4.91 ± 0.11
Phloridzin	7.30 ± 0.24
Eriodictyol	ND
Catechin	ND
Rutin	226.06 ± 21.09
Quercetin	ND
Luteolin	ND
Naringenin chalcone	ND
Kaempferol	ND

^1^Not detected.

## Data Availability

The data used to support the findings of this study are available within the article.
